# Ohmic Contacts on p-Type Al-Implanted 4H-SiC Layers after Different Post-Implantation Annealings

**DOI:** 10.3390/ma12213468

**Published:** 2019-10-23

**Authors:** Monia Spera, Giuseppe Greco, Domenico Corso, Salvatore Di Franco, Andrea Severino, Angelo Alberto Messina, Filippo Giannazzo, Fabrizio Roccaforte

**Affiliations:** 1 Consiglio Nazionale delle Ricerche–Istituto per la Microelettronica e Microsistemi, Strada VIII, n. 5–Zona Industriale, 95121 Catania, Italy; Domenico.corso@imm.cnr.it (D.C.); Salvatore.difranco@imm.cnr.it (S.D.F.); angelo.messina@st.com (A.A.M.); filippo.giannazzo@imm.cnr.it (F.G.); 2 Department of Physics and Astronomy, University of Catania, via Santa Sofia 64, 95123 Catania, Italy; 3 Department of Physics and Chemistry, University of Palermo, via Archirafi 36, 90123 Palermo, Italy; 4 STMicroelectronics, Stradale Primosole, 50, 95121 Catania, Italy; Andrea.severino@st.com

**Keywords:** ion-implantation, 4H-SiC, ohmic contacts

## Abstract

This paper reports on the electrical activation and Ohmic contact properties on p-type Al-implanted silicon carbide (4H-SiC). In particular, the contacts were formed on 4H-SiC-implanted layers, subjected to three different post-implantation annealing processes, at 1675 °C, 1175 °C, and 1825 °C. Under these post-implantation annealing conditions, the electrical activation of the Al dopant species increased from 39% to 56%. The Ti/Al/Ni contacts showed an Ohmic behavior after annealing at 950 °C. The specific contact resistance *ρ*_*c*_ could be lowered by a factor of 2.6 with the increase of the post-implantation annealing temperature. The result can be useful for application in device fabrication. Moreover, the dependence of *ρ*_*c*_ on the active acceptor concentration followed the thermionic field emission model, with a barrier height of 0.63 eV.

## 1. Introduction

Silicon carbide (4H-SiC) is an outstanding semiconductor material that offers enormous advantages with respect to silicon, in terms of energy efficiency in high-temperature, high-power and high-frequency applications [1,2,3].

One of the peculiarities of 4H-SiC is the low diffusivity of the dopant species even at high temperatures, thus making ion implantation an obligatory route for selective area doping in device fabrication [1,4]. Indeed, the major 4H-SiC devices, e.g., Schottky diodes, Junction Barrier Schottky (JBS) diodes, and metal oxide semiconductor field effect transistors (MOSFETs), are fabricated using ion-implantation doping [1].

Aluminum (Al) ion-implantation is used for the p-type doping of 4H-SiC, and the implant is typically followed by high-temperature post-implantation annealings (>1600 °C) for the electrical activation of the dopant [5,6,7]. The p-type doped regions are very important in both JBS and MOSFETs, as their electrical properties have a significant impact on the device’s performance. In this context, the Ohmic contacts formation on p-type 4H-SiC is inherently a challenging task, due to the wide band gap of the material (leading to high metal/semiconductor barrier heights) and to the high ionization energy of the Al acceptors [8]. Moreover, since the properties of Al-implanted 4H-SiC layers critically depend on the large variety of reported experimental doping and annealing conditions [9,10,11,12], understanding the behavior of Ohmic contacts on such layers remains an open issue with relevant implications for device fabrication.

Nickel films annealed above 900 °C are commonly used as an Ohmic contact on n-type 4H-SiC layers [13]. Although such nickel silicide layers exhibit an Ohmic behavior also on highly doped p-type 4H-SiC layers, their specific contact resistance *ρ*_*c*_ is typically a couple of orders of magnitude higher than on n-type 4H-SiC layers [14]. Hence, Ti/Al-related contacts, annealed at temperatures higher than 800 °C, have been proposed to form Ohmic contacts on p-type doped 4H-SiC [15,16]. These systems can give promising values of specific contact resistance *ρ*_*c*_, in the range 10^−4^–10^−5^ Ω cm^2^ [17,18]. Hence, they have been the object of several investigations aimed at understanding the role of the interface microstructure on the transition from a Schottky to an Ohmic behavior [19,20,21]. Recently, alternative solutions employing protective capping layers on Ti/Al stacks (Si [22], NiV [23], W [24], Ni [25]) or Ni-Ti-Al systems [26,27,28,29] have been proposed to limit surface degradation upon annealing and to improve the contact reliability. Among them, while Ti/Al/Ni stacks have shown promising electrical characteristics on p-type-implanted 4H-SiC [25]. However, their behavior as a function of the substrate doping, has not been studied yet.

In this paper, the electrical properties of Ti/Al/Ni Ohmic contacts on p-type-implanted 4H-SiC have been monitored for different post-implantation annealing conditions. The mechanism of carrier transport at the metal/4H-SiC interface has been attributed to the thermionic field emission, with a barrier height of 0.63 eV.

## 2. Materials and Methods

In this work, heavily doped p-type 4H-SiC layers were used. The p-type regions were created on the top of 4H-SiC (0001) n-type epitaxial layers having a nominal concentration, N_D-epi_ = 1 × 10^16^ at/cm^3^. Implantations of Aluminum (Al) ions were performed at 500 °C, using different ion energies (30–200 keV) and doses of 3 × 10^14^–1 × 10^15^ at/cm^2^ to create an almost flat profile with a thickness of about 300 nm and a concentration of 1 × 10^20^ at/cm^3^. After Al-ion implantation, the samples were protected by a graphite capping layer, created through the thermal graphitization of photoresist [17,30], and annealed under different conditions (1675 °C for 30 min, 1775 °C and 1825 °C for 15 min). Thereafter, both the implanted materials and the contacts were characterized under the electrical point of view. The electrical properties of the p-type 4H-SiC-implanted layers were determined by a combination of Van der Paw and Hall Effect measurements carried out at different temperatures. Ti(70 nm)/Al(200 nm)/Ni(50 nm) contacts were deposited by a sputtering technique (with the Ti layer in contact with the 4H-SiC) and annealed at 950 °C in an Argon atmosphere for 60 s to obtain an Ohmic behavior. Before metal deposition, the 4H-SiC surface was cleaned with a piranha solution followed by a buffered oxide etch (BOE). The surface morphology of the contacts before and after annealing was monitored by Atomic Force Microscopy (AFM), using a XE-150 microscope by PSIA (now Park Systems Corp., Suwon, Korea). Transmission Line Model (TLM) structures were fabricated by the annealing of Ti/Al/Ni pads placed at different distances (5, 10, 15, 20, and 25 μm) on a rectangular 4H-SiC area laterally isolated by trench etching. These structures were used to extract the electrical properties of the contacts [31]. The current–voltage (I–V) measurements on the TLM structures were performed on a Karl Suss Microtec probe station with a HP 4156B parameter analyzer (now Keysight technologies, Santa Rosa, CA, USA), in a four-point probe configuration.

## 3. Results and Discussion

Firstly, Van der Paw and Hall Effect measurements were carried out to determine the electrical properties of the Al-implanted 4H-SiC layers annealed at different temperatures. In particular, I–V measurements on Van der Paw structures allowed to determine the sheet resistance of p-type 4H-SiC. Then, assuming a uniform doping over the entire implanted thickness of 300 nm, the resistivity of the implanted layer (Ω cm) could be extracted. The activation energy E_A_ of the Al-implanted dopant was determined from an Arrhenius plot of the 4H-SiC sheet resistance. The values of E_A_ in the range of 99–110 meV are in agreement with typical literature values reported for these high acceptor concentrations, as well as with the theoretical relation between E_A_ and N_A_ in 4H-SiC [12,32].

Then, using this parameter, the temperature dependence of the hole concentration extracted by Hall Effect measurements could be fitted using the neutrality equation [31], determining the active acceptor concentration (N_A_), the concentration of compensating centers (N_D_) associated to residual implant defects after annealing, the percentage of an electrically active Al dopant, and the compensation ratio (N_D_/N_A_). Table I summarizes the main results of these electrical analyses carried out on the p-type-implanted 4H-SiC layers, subjected to different post-implantation annealing treatments (1675 °C, 1775 °C, and 1825 °C).

**Figure 1:**
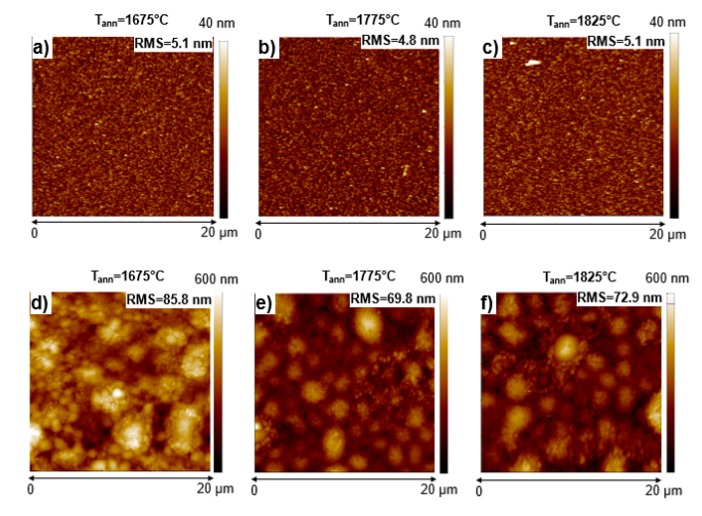
AFM images of the as deposited (**a**–**c**) and annealed (950 °C) Ti/Al/Ni contacts (**d**–**f**)) on Al-implanted silicon carbide (4H-SiC) samples, activated at three different post-implantation annealing temperatures (1675 °C, 1775 °C, and 1825 °C).

Then, Ti/Al/Ni contacts were deposited on the implanted layers. These contacts were characterized from the morphological and electrical point of view. Figure 1a–c shows the AFM images of as deposited Ti/Al/Ni contacts on p-type-implanted 4H-SiC samples, subjected to three different post-implantation annealing temperatures. Independent of the annealing process of the underlying material, the contacts have a similar morphology, with root mean square (RMS) roughness values in the range of 4.8–5.1 nm. Figure 1d–f shows the morphologies of the same Ti/Al/Ni contacts after annealing in Ar at 950 °C. Evidently, the annealing process resulted in an increase in the RMS of the contacts, due to the thermal reaction of the layer and to the presence of some metallic hillocks on the sample surface. In particular, the RMS values were 85.8 nm at 1675 °C, 69.8 nm at 1775 °C, and 72.9 nm at 1825 °C.

In fact, it has been previously reported that thermal annealing of Ti/Al/Ni layers on 4H-SiC, results in an intermixing of the metal layer, with the formation of new phases (mainly Al_3_Ni_2_), which are characterized by a high surface roughness [25].

**Figure 2:**
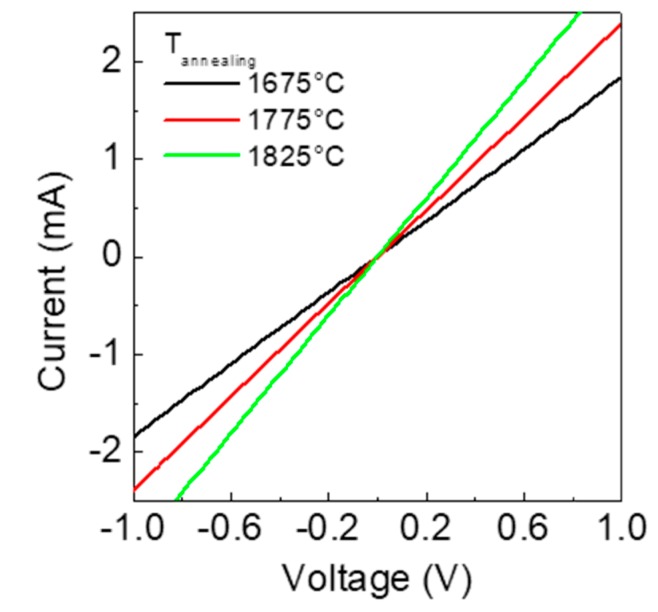
I–V curves of Ti/Al/Ni Ohmic contacts (formed at 950 °C), acquired between adjacent TLM pads at a distance of 20 µm. The Al-implanted silicon carbide (4H-SiC) samples were activated at three different post-implantation annealing temperatures (1675 °C, 1775 °C, and 1825 °C).

The electrical characterization of the contacts has been performed by I–V measurements on the fabricated TLM structures. Figure 2 shows the comparison of I–V curves acquired between adjacent contacts of the TLM structure (placed at a distance of 20 µm), for the three samples. As can be seen, linear characteristics are obtained in all three cases. Moreover, a gradual increase in the current is observed with the increase of the post-implantation annealing temperature from 1675 °C to 1825 °C.

**Figure 3:**
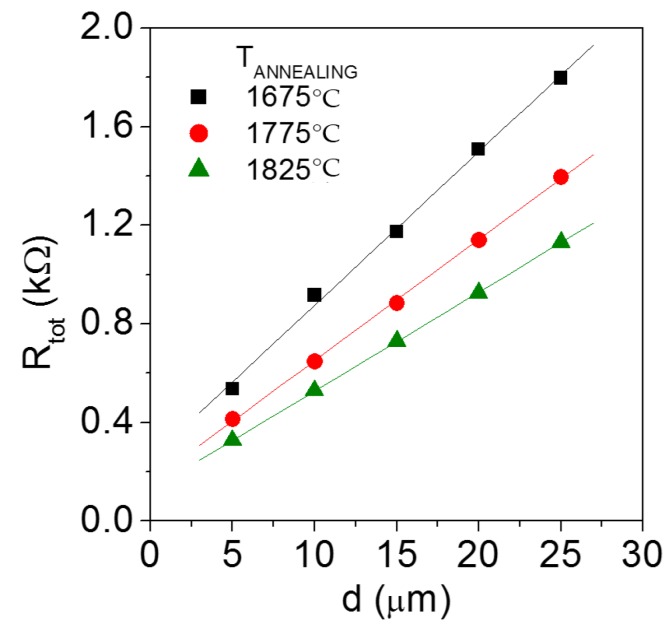
Plots of the total resistance R_TOT_ as a function of the TLM pad distance d, used for the extraction of the specific contact resistance, on Al-implanted 4H-SiC samples subjected to post-implantation annealing at T_ann_ = 1675 °C, T_ann_ = 1775 °C, and T_ann_ = 1825 °C.

From the slope of the I–V curves acquired in the three samples at different TLM pad distances, it was possible to extract the total resistance R_TOT_. Figure 3 reports the plots of the total resistance *R_TOT_* as a function of the distance *d* between the TLM pads. The different slope of *R_TOT_* in the three samples is due to the different sheet resistance of the 4H-SiC-implanted layers. In particular, the linear fits of the data gave the following values of sheet resistance of the p-type-implanted 4H-SiC: 12.4 kΩ/sq (T_ann_ = 1675 °C), 9.8 kΩ/sq (T_ann_ = 1775 °C), and 8.0 kΩ/sq (T_ann_ = 1825 °C). Such values are in agreement with those extracted by Hall measurements (see Table 1), considering the thickness of the implanted layer (300 nm) [12]. Moreover, the resistivity values are consistent with the literature data obtained for 4H-SiC layers with a similar acceptor concentration (3–6 × 10^19^ cm^−3^) [10,14,17,24,25].

The TLM analysis allowed determining the following values of the specific contact resistance: 5.2 × 10^−4^ Ω cm^2^ at T_ann_ = 1675 °C, 2.6 × 10^−4^ Ω cm^2^ at T_ann_ = 1775 °C and 2.0 × 10^−4^ Ω cm^2^ at T_ann_ = 1825 °C.

**Figure 4:**
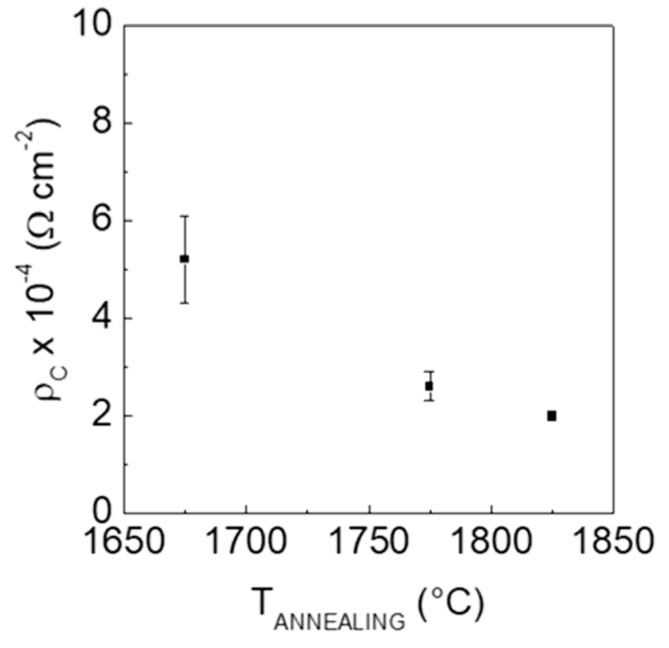
Specific contact resistance of Ti/Al/Ni contacts on p-type-implanted 4H-SiC layers as a function of the post-implantation annealing temperature.

As summarized in Figure 4, the values of *ρ*_*c*_ decrease with the increase of the post-implantation annealing temperature. This result is correlated with the improved Al activation.

To complete the electrical characterization of the Ohmic contacts, the current transport mechanism at the metal/semiconductor interface has been studied. Generally, for intermediate values of the doping concentration (10^17^ < *N_A_* < 10^19^ cm^−3^), current transport across the metal/semiconductor barrier is ruled by the *Thermionic Field Emission* (TFE) mechanism [33,34]. More specifically, the dominant current injection mechanism at the interface with 4H-SiC can be established by comparing the characteristic energy *E*_00_ with the thermal energy *kT* (where *k* is the Boltzmann constant and T is the temperature). The characteristic energy *E*_00_ is defined as:

(1) $$E_{00}=\frac{qh}{4\pi }\sqrt{\frac{N_{A}}{m^{*}\varepsilon }}$$

where *h* is the Planck constant, *ε* is the dielectric constant of 4H-SiC, m* is the tunneling effective mass, and *N_A_* is the doping concentration. In particular, for *E*_00_ ≈ *kT*, TFE represents the main transport mechanism. For the *N_A_* values in our samples (Table 1), the calculated *E*_00_ was in the range of 39–47 meV, which is comparable with *kT* of our measurement range. Hence, the temperature dependence of *ρ_c_* was described by the TFE expression:

(2) $$\rho_{c-TFE}=\frac{k\sqrt{E_{00}}\cosh\left(E_{00}/kT\right)\coth\left(E_{00}/kT\right)}{A^{*}Tq\sqrt{\pi q\left(\Phi_{B}-V_{p}\right)}}exp\left[\frac{q\left(\Phi_{B}-V_{p}\right)}{E_{00}\coth\left(E_{00}/kT\right)}+\frac{qV_{p}}{kT}\right]$$

where Φ_*B*_ is the metal/p-SiC barrier height, k is the Boltzmann constant, A* is the Richardson constant, and *V_p_* is the energy difference between the valence-band edge and the Fermi level.

**Figure 5:**
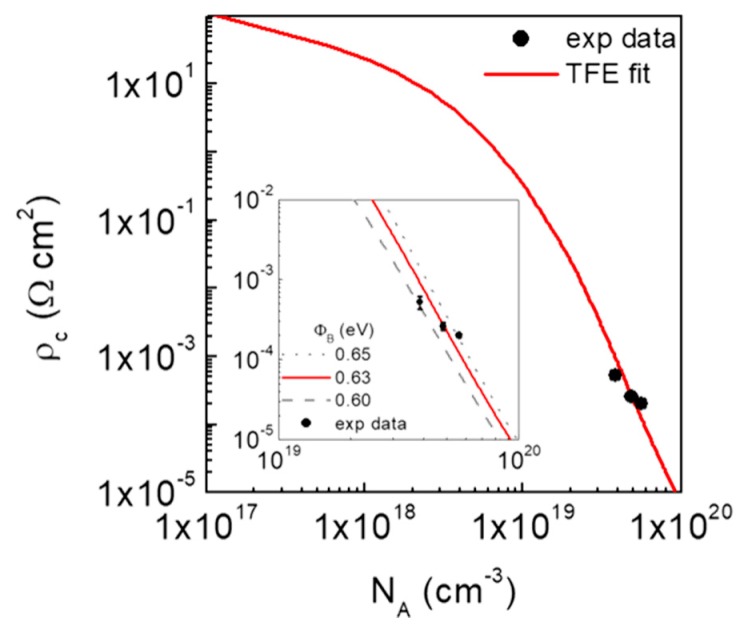
Specific contact resistance *ρ*_*c*_ of Ti/Al/Ni Ohmic contacts as a function of the acceptor concentration N_A_ in p-type-implanted 4H-SiC. The continuous line is the fit of the experimental data obtained using the TFE model with a barrier height of 0.63 eV. Inset: simulated curves for values of Φ_*B*_ = 0.60 eV, Φ_*B*_ = 0.63 eV, and Φ_*B*_ = 0.65 eV.

Figure 5 reports the experimental *ρ_c_* values as a function of *N_A_* for our three samples and the best fit of these data with Equation (2) using Φ_*B*_ as the only fitting parameter. This plot illustrates the dependence of the *ρ*_c-TFE_ function on the acceptor concentration in a wide concentration range, from 10^17^ to 10^20^ cm^−3^. For a better visualization, a plot of the data in a narrower range of *N_A_* values (10^19^–10^20^ cm^−3^) is also shown in the inset of Figure 5, together with the calculated *ρ*_*c*_ vs *N_A_* curves for values of Φ_*B*_ = 0.60 eV, Φ_*B*_ = 0.63 eV, and Φ_*B*_ = 0.65 eV. This comparison confirms that Φ_*B*_ = 0.63 eV is the barrier height value that better accounts for the experimental dependence of the contact resistance.

Other literature works described the carrier transport for Ti/Al-based contacts with the TFE model [17,19,21,24,25]. From the fit of the specific contact resistance, different values of barrier height have been obtained (see Table 2). Clearly, the value of the barrier height depends on the different experimental parameters, such as the doping, the deposition technique, the metal thickness, and the annealing conditions [35]. From Table 2, it is interesting to note that Frazzetto et al. [17] obtained a lower Φ_*B*_ of 0.46 eV using Ti/Al contacts. The lowering of the barrier has been attributed to the formation of the ternary phase Ti_3_SiC_2_ [19,20,21]. A slightly higher barrier height of 0.56 eV has been reported by Vivona et al. [25] for Ti/Al/Ni contacts (similar to our samples). In this case, an interfacial TiC layer is observed at the metal/4H-SiC interface and it has been indicated as responsible for the Ohmic behavior. An even higher barrier (0.69 eV) has been measured using Ti/Al/W [24]. In addition, barrier height values of 0.71 eV [21] or 0.82 eV [36] have been obtained using a Ti/Al scheme with different metal thickness and different annealing conditions. Hence, it is clear how the different microstructure changes occurring at the interface after thermal treatment play a key role in the value of the barrier height.

## 4. Conclusions

In conclusion, this work discussed the electrical behavior of Ohmic contacts on p-type Al-implanted 4H-SiC layers activated under different post-implantation annealings (1675 °C, 1175 °C, and 1825 °C). Ti/Al/Ni Ohmic contacts formed with a rapid annealing at 950 °C, exhibited values of the specific contact resistance in the 10^−4^ Ω cm^2^ range, which decreased with the increase of the post-implantation annealing temperature. The dependence of *ρ*_*c*_ on the active acceptor concentration is ruled by the thermionic field emission model, with a barrier height of 0.63 eV. These results can be useful to set the optimal processing conditions for Ohmic contacts on p-type regions in 4H-SiC devices.

## Figures and Tables

**Table 1:** Summary of the main electrical results obtained on p-type-implanted 4H-SiC layers, subjected to different post-implantation annealing at different temperatures (1675 °C, 1775 °C, and 1825 °C).

Annealing Temperature	Material Resistivity (Ω cm)	E_A_ (meV)	N_A_ (cm^−3^)	N_D_ (cm^−3^)	Al Activation	Compensation Ratio N_D_/N_A_
1675 °C	0.36	110	3.87 × 10^19^	3.66 × 10^18^	39%	9.4%
1775 °C	0.29	105	4.84 × 10^19^	3.49 × 10^18^	48%	7.2%
1825 °C	0.22	99	5.64 × 10^19^	3.48 × 10^18^	56%	6.2%

**Table 2:** Survey of literature results on Ti/Al-based Ohmic contacts to p-type 4H-SiC, formed under different annealing conditions. The reported barrier height values have been determined by TFE model.

Metal Scheme	Annealing Conditions	Φ_*B*_ (eV)	Ref.
Ti_(100 nm)_/Al_(300 nm)_	950 °C (1 min, Ar)	0.46	[17]
Ti_(30 nm)_/Al_(70 nm)_	1000 °C (1 min, Ar)	0.71	[21]
Ti_(70 nm)_/Al_(200 nm)_/Ni_(50 nm)_	950 °C (1 min, Ar)	0.56	[25]
Ti_(70 nm)_/Al_(200 nm)_/W_(50 nm)_	1100 °C (1 min, Ar)	0.69	[24]
Ti_(30 nm)_/Al_(100 nm)_/NiV_(25 nm)_	1000 °C (2 min, Ar)	0.43	[19]
Ti_(70 nm)_/Al_(200 nm)_/Ni_(50 nm)_	950 °C (1 min, Ar)	0.63	This work
